# Peroral endoscopic myotomy to treat epiphrenic diverticulum: a step-by-step video demonstration

**DOI:** 10.1055/a-2106-1183

**Published:** 2023-06-27

**Authors:** Apostolis Papaefthymiou, Benjamin Norton, Andrea Telese, Nasar Aslam, Vinay Sehgal, Rehan Haidry

**Affiliations:** Endoscopy Unit, University College London Hospitals (UCLH), London, United Kingdom

Epiphrenic diverticula represent an uncommon cause of dysphagia. Surgical diverticulectomy with myotomy has been the standard therapeutic approach, providing a high rate of short- and long-term improvement, albeit with a high morbidity rate 1 .

An 84-year-old woman with a 2-year history of dysphagia was referred to our unit due to an epiphrenic diverticulum and she was offered peroral endoscopic myotomy (POEM). A large epiphrenic diverticulum (approximately 10 cm) was identified in endoscopy. It was filled with a significant amount of food and there was a narrowing of the esophageal lumen ( Video 1 ). The solid residue was removed to clear the operational view and a guidewire was left in the stomach for safety. An indigo carmine and adrenaline solution was injected over the anterior aspect of the septum followed by a vertical mucosal incision. Careful injection and dissection of the narrow submucosa on the pouch side was performed to separate the mucosal layer of the pouch from the septum, followed by the same tunneling process in the contralateral esophageal side up to the level of the cardia. The distal margin of the tunnel was confirmed by retroflexion. Afterwards, the thick muscular septum was dissected and the muscle located on side of the pouch was carefully divided. A full thickness myotomy was carried out from the first 2 cm distal to the base of the pouch, followed by a partial myotomy of the circular fibers extending into the cardia ( Fig. 1 ). Bleeding points were coagulated with coagulation forceps. Finally, gentamicin was infused into the tunnel and the mucosal incision was closed with clips. No adverse events were recorded and the patient reported significant symptom improvement.

**Video 1** A step-by-step peroral endoscopic myotomy to treat an epiphrenic diverticulum.


**Fig. 1:**
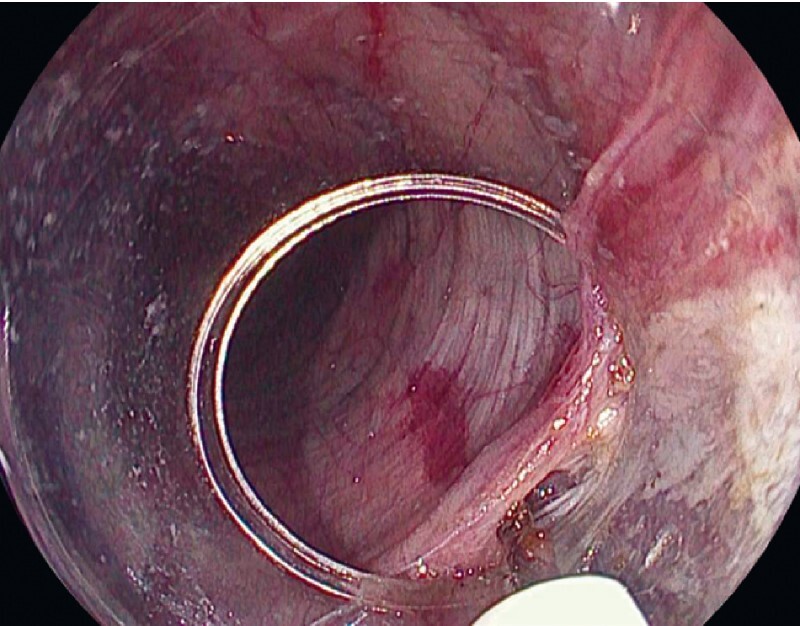
Full thickness myotomy of the lower esophageal muscular fibers.

The introduction of POEM into clinical practice has changed the therapeutic approaches to esophageal disorders, with emerging data reporting a success rate of 94.2 % (87.3 %−100 %) 2 in treating epiphrenic diverticula with safety 3 . Long-term follow-up studies are warranted to assess its efficacy compared to the established methods.

Endoscopy_UCTN_Code_TTT_1AO_2AG

## Notes

Endoscopy E-Videos

https://eref.thieme.de/e-videos
*E-Videos* is an open access online section of the journal *Endoscopy* , reporting on interesting cases and new techniques in gastroenterological endoscopy. All papers include a high-quality video and are published with a Creative Commons CC-BY license. Endoscopy E-Videos qualify for HINARI discounts and waivers and eligibility is automatically checked during the submission process. We grant 100% waivers to articles whose corresponding authors are based in Group A countries and 50% waivers to those who are based in Group B countries as classified by Research4Life (see: https://www.research4life.org/access/eligibility/ ). This section has its own submission website at https://mc.manuscriptcentral.com/e-videos
